# Empowering Communities of Color for Environmental Health and Justice: The Stand Together Against Neighborhood Drilling in Los Angeles Case

**DOI:** 10.5888/pcd21.230248

**Published:** 2024-02-22

**Authors:** Jason A. Douglas

**Affiliations:** 1 University of California, Irvine, Department of Health, Society, and Behavior and the Center for Environmental Health Disparities Research, Irvine, California

Environmental conditions such as the air quality where we live, learn, play, and work contribute to community health and well-being (1). Notably, air pollution has declined significantly in the US. For example, concentrations of fine particulate matter (PM^2.5^) produced by fossil fuel combustion decreased by 40.4% between 2000 and 2016 (2). Yet communities of color (eg, Black and Latiné communities) experience a persistent burden of air pollution exposure. In 2016, PM^2.5 ^concentrations in majority Black communities were 13.7% higher than in majority White communities, and PM^2.5^ is a known cause of chronic diseases such as cardiovascular disease and asthma (2,3). Furthermore, Black Americans experience an unequal burden of cardiovascular risk factors such as hypertension and asthma and are 30% more likely to die from cardiovascular disease than White Americans (4).

Disparities in environmental exposures and resultant chronic disease are a product of environmental racism, which is a system of discriminatory policies and practices that systematically and unevenly distribute political power and access to resources to the more privileged members of society (5). Residential segregation is a poignant example of environmental racism in action. During the 1930s New Deal era, the Home Owners’ Loan Corporation mapped communities along racial and ethnic lines to identify neighborhoods for emergency home lending (6). Communities with high densities of people of color and foreign-born residents, and neighborhoods proximal to known sources of environmental pollution, were deemed unsuitable for federal backing of home loans (6). These policies isolated communities of color and inhibited the advancement of generational wealth and community health (5,6).

Spanning time and space, communities redlined in the 1930s are present-day geographies of environmental health disparities, which are unjust and avoidable disparities in health and chronic disease arising from uneven social and environmental conditions. For example, the available evidence from California indicates that 1) oil and gas wells contribute to significant increases in PM^2.5 ^concentration and 2) Black communities are disproportionately exposed to oil and gas wells (7,8). In contrast, Black and broader communities of color often lack critical environmental resources, such as public parks and urban forests, that are associated with the reduction of air pollution and chronic disease (6,9). This amalgam of inequities places communities of color in triple jeopardy of disproportionate environmental exposures, unequal access to health-promoting resources, and resultant environmental health disparities (9).

Persistent patterns of environmental racism must be addressed to reduce the burden of environmental health disparities. Unfortunately, public health measures that have traditionally targeted individual-level behavior change are ill-equipped to promote health equity in this context (10). Instead, addressing environmental health disparities requires coordinated efforts that reduce environmental exposures and promote health equity (10). In this essay, I present a grassroots approach to reducing environmental health disparities through community capacity-building and empowerment strategies.

## Empowering Communities for Environmental Health and Justice

A growing body of literature suggests that community participation in research, action, and governance is an effective community empowerment strategy for redressing environmental health disparities (10,11). The Communities for a Better Environment (CBE) Process Model for Structural Change (Figure) illustrates an iterative, nonlinear capacity-building process that integrates community organizing and community-based participatory research to inform and advance legal strategies and policies that address environmental racism (10).

**Figure:**
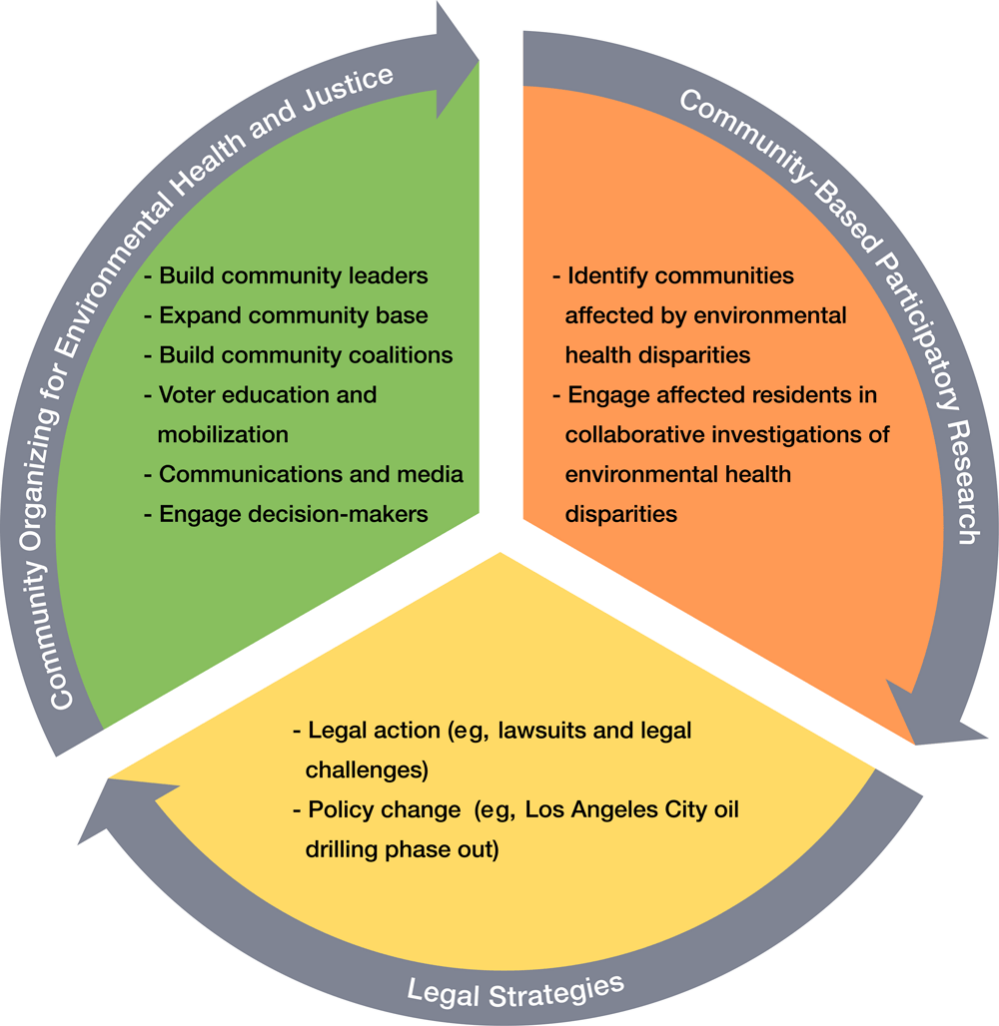
Communities for a Better Environment Process Model for Structural Change.

Community organizing mobilizes historically marginalized communities, community-based organizations, and allies with aligned interests and concerns to build community capacity and exercise power toward achieving structural changes that promote community health (10). In community organizing, capacity building is a central process that entails building community leaders empowered to drive community organizing initiatives that address the interests of affected residents (eg, reducing environmental exposures). Community leaders empowered with a working knowledge of environmental health disparities can then expand the community base by sharing their knowledge with residents affected by environmental health disparities and encouraging resident participation in further community organizing initiatives. The CBE model also involves building coalitions of aligned organizations that can pool resources toward advancing health equity. As such, pooling resources across coalitions can increase the capacity of community leaders and their respective organizations to work collaboratively on voter education and mobilization efforts that engage residents in developing an understanding of the impact of environmental racism and how to amplify community voices by engaging in electoral systems. Community coalitions have also made inroads into voicing the concerns of affected residents through communications and media, wherein residents develop their own stories and create strong and effective messages for key audiences. Critically, these capacity-building efforts empower residents to engage decision-makers by providing testimony at town hall meetings, supervisory board meetings, and other political forums to voice their interests and concerns (10).

Community-based participatory research (CBPR) dovetails with community efforts to reduce the burden of chronic disease and advance social, environmental, and racial justice by providing a systematic mode of inquiry involving people directly affected by the issue of study (11). CBPR affords inroads for the co-construction of knowledge, whereby all participants (eg, community youths, leaders, researchers) contribute to the research, offering their situated knowledge and expertise throughout the process. Thus, by using rigorous research methods to work with residents and broader coalitions to collaboratively identify affected communities (eg, communities disproportionately burdened by oil and gas wells) and examine environmental health disparities based on the interests and perspectives of affected residents (11), residents, community organizers, and broader community coalitions can then leverage empirical data to demand structural solutions through legal actions and policy changes with the potential to redress decades of persistent environmental health disparities (Figure).

## The Stand Together Against Neighborhood Drilling Case

Large-scale coalitions that use community organizing and CBPR have made promising progress toward redressing environmental racism. For example, Stand Together Against Neighborhood Drilling (STAND-L.A.) — a coalition of 7 community-based organizations advocating for environmental justice in Los Angeles, California — came together to address unequal exposure to air-polluting oil and gas wells in communities of color. STAND-L.A. engaged in a process that closely aligns with the CBE Process Model for Structural Change (Figure).

Motivated by resident concerns around the Los Angeles oil drilling context, the STAND-L.A. coalition leveraged its organizing and research capacity to establish a group of community leaders empowered with a situated understanding of the public health effects of oil and gas wells. STAND-L.A. community leaders worked with affected residents to expand their community base by sharing their knowledge of environmental health disparities related to oil and gas wells. Community leaders also steered voter education and mobilization efforts. They developed communications and media, such as the *No Drilling Where We’re Living: The Fight Against Big Oil in LA Neighborhoods* campaign video (12), to disseminate CBPR findings, resident perspectives, and campaign accomplishments and suggest policy solutions. Simultaneously, STAND-L.A. leveraged its shared resources to investigate the effects of oil drilling in 9 Los Angeles communities. Its research revealed that 70% of the 1,071 new and active oil wells in Los Angeles City as of 2015 were within 1,500 feet of homes or sensitive land use areas such as schools and hospitals (13). Subsequent research documented significant health effects for residents within 2,500 and 3,200 feet of new and active oil wells, which are disproportionately located in communities of color that lack greenspace and are at increased risk for chronic disease and broader health disparities because of these environmental exposures (7–9,13,14). For example, residents of Wilmington, California — a large, majority Latiné community that is low-income, greenspace-poor, and air pollution–burdened — have the highest cancer risk in Southern California, with more than 1,000 excess cancer cases per million residents (13,14). With the power of community in one hand and science in the other, STAND-L.A. was empowered to build community capacity and power to highlight the true root causes of resident exposures to oil and gas wells and the environmental health disparities that resulted from these exposures. This capacity-building campaign, which spanned nearly 10 years, placed environmental justice communities in a position to engage with decision-makers in town hall meetings to share research and residents’ interests and take legal action in the form of lawsuits against the City of Los Angeles over racially discriminatory oil drilling permitting (14). As a result, in December 2022 and January 2023, the City and County of Los Angeles, respectively, passed ordinances to phase out oil drilling (14). These historic policy-change wins, which begin to provide health and safety protections that reduce the burden of environmental health disparities for underserved Los Angeles residents who have endured air pollution exposure inequities for decades, were ultimately achieved by building community capacity and power to produce structural changes that attend to the voices and interests of affected residents.

## Conclusions

Without universal policies and protections that address the inequities produced and reproduced by environmental racism, communities of color continue to bear an uneven burden of environmental health disparities (5,6). Federal agencies such as the National Aeronautics and Space Administration (NASA) are increasingly calling for community engagement strategies to inform new approaches for advancing environmental justice (15). CBE’s Process Model for Structural Change builds on decades of evidence from the field to find a path forward that engages affected communities to 1) identify the most pressing environmental health disparities they face and 2) build community capacity and power to examine and address environmental health disparities through sustained cooperative partnerships and advocacy efforts (10,11). In closing, I argue that communities affected by environmental health disparities are best suited to develop solutions that advance health and well-being and promote environmental justice where we live, learn, play, and work.
